# Alterations in heart rate variability during everyday life are linked to insulin resistance. A role of dominating sympathetic over parasympathetic nerve activity?

**DOI:** 10.1186/s12933-016-0411-8

**Published:** 2016-06-28

**Authors:** Maria K. Svensson, Stina Lindmark, Urban Wiklund, Peter Rask, Marcus Karlsson, Jan Myrin, Joel Kullberg, Lars Johansson, Jan W. Eriksson

**Affiliations:** Department of Medical Sciences, Uppsala University Hospital, 751 85 Uppsala, Sweden; Department of Medicine, Umeå University Hospital, Umeå, Sweden; Department of Biomedical Engineering & Informatics, Umeå University Hospital, Umeå, Sweden; Department of Clinical Physiology, Örebro University Hospital, Örebro, Sweden; Department of Radiology, Uppsala University Hospital, Uppsala, Sweden

**Keywords:** Heart rate variability, Spectral analysis, Autonomic nervous system, Insulin sensitivity, Type 2 diabetes

## Abstract

**Aims:**

To evaluate the role of the autonomic nervous system (ANS) in the development of insulin resistance (IR) and assess the relationship between IR and activity of ANS using power spectrum analysis of heart rate variability (HRV).

**Subjects and methods:**

Twenty-three healthy first-degree relatives of patients with type 2 diabetes (R) and 24 control subjects without family history of diabetes (C) group-matched for age, BMI and sex were included. Insulin sensitivity (M value) was assessed by hyperinsulinemic (56 mU/m^2^/min) euglycemic clamp. Activity of the ANS was assessed using power spectrum analysis of HRV in long-term recordings, i.e., 24-h ECG monitoring, and in short-term recordings during manoeuvres activating the ANS. Computed tomography was performed to estimate the amount and distribution of abdominal adipose tissue.

**Results:**

Insulin sensitivity (M value, mg/kg lbm/min) did not differ significantly between the R and C groups. Total spectral power (P_tot_) and very low-frequency (P_VLF_) power was lower in R than C during 24 h ECG-recordings (p = 0.02 and p = 0.03). The best fit multiple variable linear regression model (r^2^ = 0.37, p < 0.001 for model) indicated that body composition (BMI) and long-term low to high frequency (LF/HF) power ratio (std β = −0.46, p = 0.001 and std β = −0.28, p = 0.003, respectively) were significantly and independently associated with the M value.

**Conclusion:**

Altered heart rate variability, assessed by power spectrum analysis, during everyday life is linked to insulin resistance. The data suggest that an increased ratio of sympathetic to parasympathetic nerve activity, occurring via both inherited and acquired mechanisms, could potentially contribute to the development of type 2 diabetes.

**Electronic supplementary material:**

The online version of this article (doi:10.1186/s12933-016-0411-8) contains supplementary material, which is available to authorized users.

## Background

Insulin resistance (IR) is associated with future risk of developing type 2 diabetes (T2D). As a group, relatives of patients with T2D are more insulin-resistant and also have an increased risk of developing T2D later in life but there is an overlap in IR when compared to subjects without a family history of T2D [1]. The pathophysiological mechanism(s) causing IR are still not fully understood, but candidate genes related to cellular insulin action may be potential contributors [1–3]. The fact that cellular insulin resistance may be reversible [4, 5] indicates that the in vivo milieu is of importance and for example enhanced activity in the sympathetic nervous system (SNS) has been suggested as a mediator of IR [6]. Elevated activity of the SNS has thus been proposed as a causal factor in the “insulin resistance syndrome” [7]. Not only an elevated activity of the SNS, but also an attenuated activity in the parasympathetic nervous system could contribute and we have previously reported an association between IR and reduced parasympathetic-to-sympathetic activity in non-diabetic subjects [8]. This is in line with previous findings that non-diabetic offsprings of patients with T2D have an altered response to acute hyperinsulinemia with an increase in total spectral power and low-frequency to high-frequency (LF/HF) power ratio and a decrease in high-frequency power HRV [9]. A link between HRV and IR has also been found in other populations [10–12].

The main objective of this study was to further assess the relationship between IR and activity of the ANS using power spectrum analysis of HRV analysed from long-term, 24-h ECG-recordings, obtained during normal everyday life. This was done in healthy non-diabetic subjects. We also compared first-degree relatives of patients with T2D with subjects without a family history of T2D. In addition, power spectrum analysis of HRV was assessed using short-term recordings both during resting conditions and during manoeuvres that activates the sympathetic or the parasympathetic nervous system [8, 13].

## Subjects and methods

### Subjects

Forty-seven healthy, non-diabetic subjects were enrolled. Twenty-three subjects had either two first-degree or one first-degree plus at least two second-degree relatives with T2D (R) and 24 subjects had no known family history of T2D (C). The R and C groups were group-matched for sex, age and BMI. Seven of the enrolled subjects had taken part in a previous study [8] and were thus assessed at two different time-points in years 1997–98 and 2004, mean 6 years apart. The subjects were recruited by advertisement in the local newspaper. The participants were healthy and had no signs of diabetes or pre-diabetes, systemic chronic diseases, infections or inflammation as assessed by medical history and blood chemistry. Informed consent was obtained from all subjects and the study protocol was approved by the Ethical Committee of Umeå University.

### Protocol

The examinations were performed at four different visits within 4 weeks and in the following sequence: blood sampling, OGTT and questionnaire, euglycemic hyperinsulinemic clamp, short- and long-term HRV and computed tomography (CT). All examinations except the long-term HRV and the CT were performed in the morning after an overnight fast (>10 h). *Lean body mass* was determined by an bioelectrical impedance analysis method (BIA 101-Fitness RJL Systems, Detroit, MI, USA) [14]. *Oral glucose tolerance test* (OGTT) was performed with the subjects in the sitting position when ingestion of 75 g glucose in a liquid solution and venous blood samples for analyses of blood glucose and serum insulin were then obtained in the fasting state and after 120 min.

### Questionnaire to assess self-rated stress behaviour

The Everyday Life Stress Scale instrument [15] was used to assess the level of self-rated stress behaviour. The instrument consists of two major themes, i.e., time urgency/impatience and easily aroused irritation/hostility. The responses to the 20 different statements were given using a four-point scale (0–3) where higher scores indicate more stressful reactions.

### Euglycemic hyperinsulinemic clamp

Insulin sensitivity was assessed using the euglycemic hyperinsulinemic clamp technique as previously described [8]. The rate of glucose infusion served as a measure of insulin sensitivity and the M value was calculated by dividing the amount of glucose infused during the last 60 min (steady state) of the clamp by lean body mass (mg/lbm kg/min). The insulin sensitivity index (ISI) was calculated by dividing the M value by the mean insulin concentration during the same period of the clamp (100 × mg/kg/min/[mU/L]) [16].

### Computed tomography (CT)

Forty-four of the 47 subjects (23 R, 21 C) did the performed CT examinations/scans and they were examined in the evening (non-fasting state) using the Siemens Somatom Plus 4 CT system, version VB 40c (Siemens, Erlangen, Germany), 140 kV, FoV 352, mAs 180, slice thickness 10 mm, fixed filtration. The CT-scans were obtained in the lumbar region, at the L2 and L4 level. Adipose tissue was determined as Hounsfield values between −190 and −30 HU from the L4 slice. Abdominal subcutaneous (SAT) and visceral adipose tissue (VAT) was separated by manual segmentation [17] using software ImageJ [18]. Fat infiltration of the liver (liver fat) was determined from the L2 slice. Liver tissue was also segmented manually and the mean Hounsfield value was used.

### Heart rate variability (HRV) assessments

*A short*-*term recording of HRV* was performed as previously described in detail [19]. *Power spectrum analysis* was performed using auto-regressive modelling of linearly detrended heart rate data [20].

A *long*-*term HRV recording* was performed during normal day-time activities, night-time and sleep. We used 24-h ECG monitoring (Holter), with a digital recorder unit (Braemer DL 700, Braemer Inc. Burnsville, MN, USA) and the recording was then analysed using a PC-based Holter system (Aspect Holter System, GE Healthcare, Borlänge, Sweden). After manual editing, R–R intervals and the classification of heartbeats were analysed using Matlab (Mathworks Inc., Natick, Mass., USA) [21]. Undetected ectopic beats were removed by an algorithm for automatic filtering [21, 22]. Power spectrum analysis was performed by fast Fourier transformation (FFT) of R–R intervals related to normal interbeat intervals [21]. HRV was only analysed in recordings with more than 70 % of registration time (24 h). Five subjects were not included in the HRV analyses, three had recordings <70 % of the registration time, one subject had atrial fibrillation and in one subject the technical quality of the recording was poor and not sufficient to support the analysis. Taken together power spectrum analysis of long-term HRV was performed in 43 subjects (21 R and 22 C). The HRV indices (see Table 2) were calculated as average data over 24 h.

*Power spectrum analysis* was performed as total spectral power *(P*_*tot*_) and the power of the very low-frequency (VLF; 0.003–0.04 Hz), low-frequency (LF; 0.04–0.15 Hz) and high-frequency (HF; 0.15–0.50 Hz). The components were calculated and log-transformed. The variability of the HF component is mainly mediated by parasympathetic activity, whereas the LF fluctuations are mediated by both sympathetic and parasympathetic activity [23]. The LF to HF power ratio was used as an indicator of the balance between sympathetic and parasympathetic modulation of the heart rate.

The time-domain parameters SDNN, SDNNindex, SDANN, RMSSD, pNN50, SD1 and SD2 were also calculated. SDNN is the standard deviation of all NN intervals. SDANN is the standard deviation of the consecutive 5-min averages of NN intervals. SDNNindex is the mean of the 5-min standard deviation of the NN intervals. RMSSD is the root mean square of successive differences of NN intervals. pNN50 is the proportion of differences in consecutive NN intervals >50 ms divided by the total number of NN intervals. SD1 and SD2 are scatterplots of each R–R interval against the preceding R–R interval and Poincaré plots were constructed [21]. SD1 and SD2 are two time-domain parameters calculated from Poincaré plots, i.e., scatterplots of each R–R interval against the consecutive value and are measures of the standard deviation in two perpendicular directions in the plots. SD1 describes the magnitude of the beat-to-beat variability, reflecting vagal modulation of HRV and is strongly correlated with the HF spectral component. SD2, on the other hand, describes the fluctuations in mean R–R interval over the 24-h period. Data on SD1 and SD2 are presented in Table 3. Calculations were performed using the Matlab Software (MathWorks, Natick, Mass., USA).

### Blood chemistry

Blood glucose concentrations were determined by HemoCue glucose system (HemoCue AB, Ängelholm, Sweden) and HbA1c by high-pressure liquid chromatography (HPLC) (Integral 4000, BioRad, Anaheim, California, USA). Serum insulin concentrations were measured by micro particle immuno assay (MEIA) (Abbot Imx, Abbot Laboratories, Abbot Park, Illinois, USA). All other analyses of blood chemistry were performed according to routine methods at the Department of Clinical Chemistry, Umeå University Hospital.

### Statistical methods

Data are mean ± SD or as indicated. Differences between groups were tested using Students unpaired t test. Analyses of data from the short-term HRV examination were performed using ANOVA of repeated measurements. Analysis of variance (ANCOVA) with age as covariate was used to analyse data from the long-term HRV examination. Intergroup comparisons were performed by including a binary variable in the ANCOVA, whereas the relation between HRV and IR was investigated by including the M value as covariate in the ANCOVA. Univariate regression analyses were utilised to demonstrate associations between M value and long-term HRV parameters. Long-term HRV parameters that were near-significantly associated with M value in the univariate analyses (p ≤ 0.1) were included in multivariate analyses one by one adjusting for age and body composition (as indicated) (see Table 3 and Additional file 1: Table S3). Multivariate regression analyses (the enter statistics in SPSS) were used to assess independent relationships between M value, age and body composition, and long-term HRV parameters. p values less than 0.05 were considered as statistically significant. Statistical analyses were performed using the SPSS software package version 18 (SPSS Inc., Chicago, IL).

## Results

### T2D relatives (R) and control subjects (C)

**Fig. 1:**
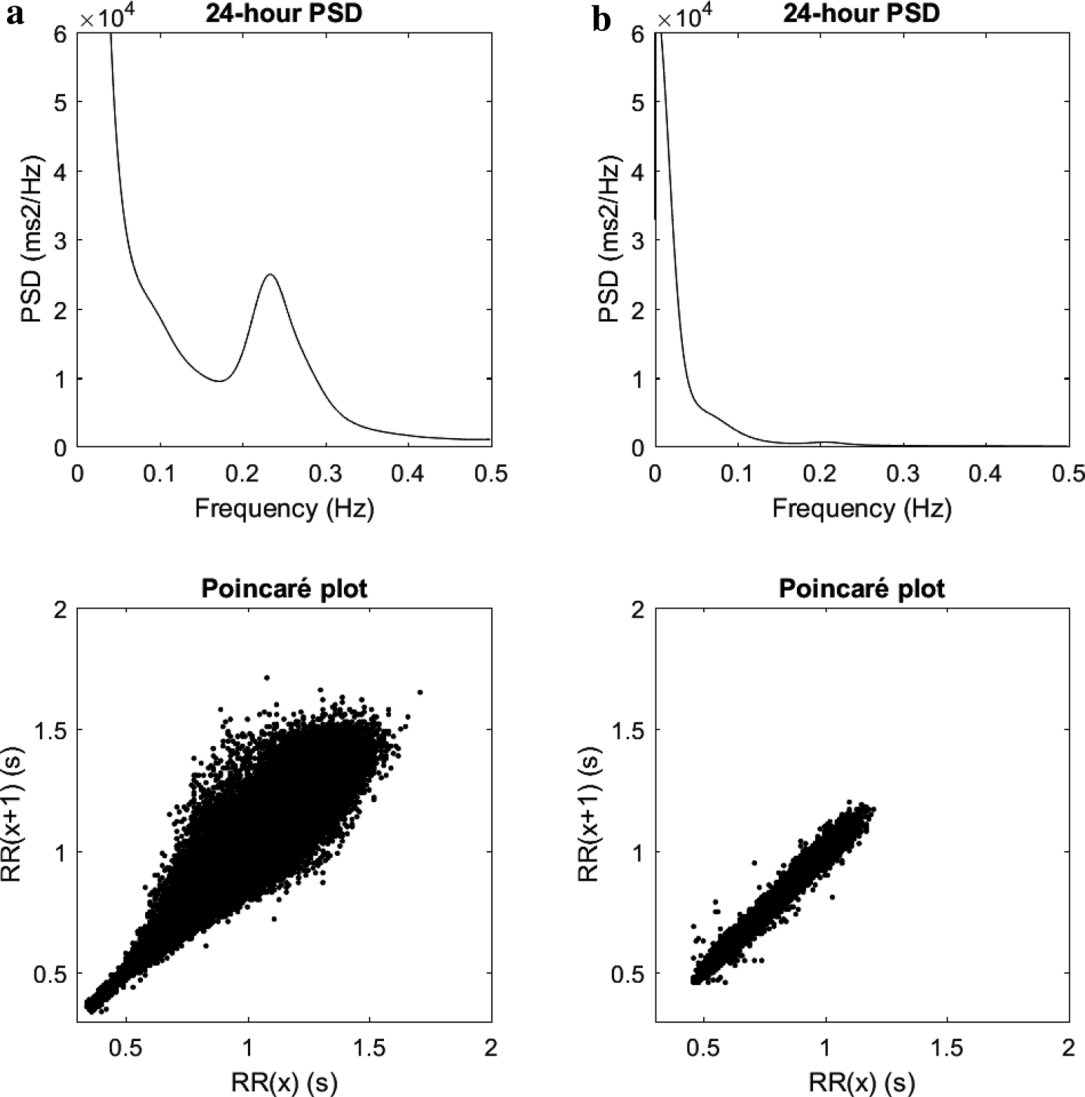
Long-term heart rate variability (HRV) using 24-h ECG recordings in two subjects where the subject to the *right* (**a** control) presented higher heart rate variability compared to the subject shown to the *left* (**b**). *Top panels* show the total average power spectrum and the *bottom panels* show Poincaré plots where each point shows the relation between two subsequent RR intervals. 24 h PSD = average power spectral density over 24-h, RR(x) = “current” RRinterval, RR(x−1) = previous RR-interval

T2D relatives (R) and control subjects (C) groups were matched for sex, age and BMI as shown in Table 1. There were no significant differences between the groups except that the 2-h blood glucose levels during OGTT was slightly higher in R (p = 0.049). No differences in the Everyday Life Stress Scale instrument were found between R and C and there was no association between IR and level of self-rated stress behaviour overall (not shown). During the short-term HRV assessments, there was a tendency towards a higher LF to HF power ratio in R (Additional file 1: Table S1). During the 24-h ECG recordings the frequency domain indices age-adjusted total spectral power, VLF power and SD1 were significantly lower in R compared to C as displayed in Table 2 and illustrated in Fig. 1, where one of the control subjects (C), to the left, displays a normal HRV compared to a T2D relative (R) who has a reduced HRV. In parallel, the time domain indices SDNN, SDNNindex, RMSSD and pNN50 were also significantly lower in R compared to C as displayed in Table 2.

**Table 1:** Clinical and biochemical characteristics of type 2 diabetes relatives (R) and control subjects (C)

	Relatives (R) n = 23	Controls (C) n = 24	p value
Sex (m/f)	12/11	13/11	n.s.
Age (years)	46.8 (12.0)	47.2 (11.7)	n.s.
BMI (kg/m^2^)	25.1 (3.8)	25.0 (3.1)	n.s.
Weight (kg)	78.4 (16.4)	77.5 (15.1)	n.s
Waist (cm)	88.6 (12.0)	88.2 (11.5)	n.s.
Waist/hip ratio	0.86 (0.09)	0.85 (0.09)	n.s.
Heart rate at rest (beats/min)	65.7 (8.1)	66.3 (7.5)	n.s.
Systolic blood pressure (mmHg)	118 (18)	113 (11)	n.s.
Diastolic blood pressure (mmHg)	77 (12)	79 (13)	n.s.
Smokers/non-smokers	1/22	3/24	n.s.
Fat mass (%)	28.2 (7.2)	28.8 (7.3)	n.s.
Visceral adipose tissue (VAT, cm^2^)^a,b^	94.1 (56.4)	101.5 (56.2)	n.s.
Subcutaneous adipose tissue (SAT, cm^2^)^a,b^	204.4 (75.0)	220.0 (83.1)	n.s.
Visceral/subcutaneous adipose tissue ratio^a,b^	0.48 (0.28)	0.49 (0.28)	n.s.
Liver fat content (Mean HU)	66.0 (6.4)	64.2 (5.9)	n.s.
HbA1c (IFCC; mmol/mol)^c^	34.1 (4.0)	33.7 (8.0)	n.s.
Fasting blood glucose (mmol/L)	5.5 (0.6)	5.5 (0.5)	n.s.
Fasting serum insulin (mU/L)	7.5 (6.9)	6.9 (3.6)	n.s.
HOMA IR	1.8 (11.7)	1.7 (50.9)	n.s.
HOMA β %	81.0 (69.5)	80.1 (41.2)	n.s.
OGTT 2 h blood glucose (mmol/L)	7.3 (1.3)	6.5 (1.3)	0.049
Serum cholesterol (mmol/L)	5.1 (0.9)	5.2 (0.9)	n.s.
Serum HDL-cholesterol (mmol/L)	1.36 (0.38)	1.50 (0.43)	n.s.
Serum LDL-cholesterol (mmol/L)	3.2 (0.9)	3.2 (0.8)	n.s.
Serum triglycerides (mmol/L)	1.21 (0.73)	1.07 (0.39)	n.s.
M value (mg/kg lean body mass/min)	12.4 (5.0)	13.2 (4.5)	n.s.
Mean serum insulin during clamp (mU/L)	92.9 (31.4)	94.6 (22.0)	n.s.
Insulin sensitivity index (ISI) [(100 × mg/kg/(mU/L)]	15.0 (7.2)	15.0 (7.0)	n.s
Plasma NEFA (µmol/L)			
Basal	0.28 (0.10)	0.33 (0.11)	0.09
During clamp	0.03 (0.05)	0.02 (0.01)	n.s.

Data are means (sd) or number of subjects

*HU* hounsfield units

^a^Only subjects participating in the CT examination (n = 44; 21 R. 23 C)

^b^Calculated values obtained from the CT scans at the L2 and L4 levels

^c^Calculated values from Mono S

**Table 2:** Heart rate variability (HRV) frequency-, time- and poincaré parameters from the long-term 24-h ECG recordings during everyday life and daily activities in T2D relatives (n = 21) and control subjects (n = 21)

	Relatives (n = 21)	Controls (n = 21)	p value
Frequency domain indices			
P_tot_ (ms^2^, log)	3.50 (0.16)	3.61 (0.25)	0.02
P_VLF_ (ms^2^, log)	3.28 (0.15)	3.38 (0.21)	0.03
P_LF_ (ms^2^, log)	2.96 (0.23)	3.06 (0.32)	0.09
P_HF_ (ms^2^, log)	2.47 (0.28)	2.58 (0.43)	0.10
P_LF_/P_HF_ (log)	0.49 (0.17)	0.48 (0.26)	0.74
Time domain indices			
SDNN	151 (24)	169 (45)	0.09
SDANN (ms)	137.8 (25.8)	150.7 (42.3)	0.21
SDNNindex	60.9 (11.0)	72.1 (21.4)	0.008
RMSSD (ms)	34.0 (11.3)	44.8 (22.1)	0.02
pNN50 (%)	10.0 (5.75)	16.2 (11.7)	0.006
Poincaré indices			
SD1 (ms)	22.9 (6.7)	29.2 (13.8)	0.01
SD2 (ms)	212 (34)	235 (61)	0.09
SD1/SD2	0.11 (0.03)	0.12 (0.04)	0.08
RR interval (s) (average heart rate)	0.78 (0.08)	0.83 (0.08)	0.06

Five subjects missing, see methods section. Values are mean values and standard deviation (sd). Spectral indices are log-transformed. p values are derived from a comparison of relatives and controls analysis of variance (ANCOVA) of repeated measurements, adjusting for age. *HRV* heart rate variability, *P*_*tot*_ total power, *P*_*VLF*_ power of very low frequency component, *P*_*LF*_ power of low frequency component, *P*_*HF*_ power of high frequency component, *P*_*LF*_ */P*_*HF*_ power of low frequency/power of high frequency ratio, *SDNN* is the standard deviation of all NN intervals, *SDANN* is the standard deviation of the consecutive 5-min averages of NN intervals, *SDNNindex* is the mean of the 5-min standard deviation of the NN intervals, *RMSSD* is the root mean square of successive differences of NN intervals, *pNN50* is the proportion of differences in consecutive NN intervals >50 ms divided by the total number of NN intervals, *SD1* and *SD2* are two time-domain parameters calculated from Poincaré plots, i.e., scatterplots of each R–R interval against the consecutive value, *SD1* describes the magnitude of the beat-to-beat variability, *SD2* describes the fluctuations in mean RR interval over the 24-h period

### Short-term heart rate variability, insulin sensitivity and body composition

In the short-term HRV examination M value was significantly associated to LF to HF power ratio (p = 0.007). In univariate regression analyses for each part of the short-term HRV examination there were significant and positive associations between M value and HF power, normalised LF power and LF to HF power ratio during the cold pressor test (r = 0.33, p = 0.036, r = −0.35, p = 0.0.26 and r = −0.37 p = 0.019). In univariate analyses of short-term HRV in all subjects VAT was inversely associated with total and HF power both in the basal state (r = −0.38, p = 0.014 and r = −0.420, p = 0.007) and during controlled breathing (r = −0.43, p = 0.006 and r = −0.46, p = 0.003). SAT was inversely associated with HF power (r = −0.36, p = 0.023) and near-significantly with total and LF power during controlled breathing (r = −0.30, p = 0.06 and r = −0.28, p = 0.08, respectively). BMI was inversely significantly associated with HF power during controlled breathing (r = −0.35, p = 0.023).

### Long-term heart rate variability, insulin sensitivity and body composition

In all subjects the M value was negatively and significantly associated with BMI (r = −0.54, p < 0.001), subcutaneous (SAT) (r = −0.39, p = 0.009) and visceral adipose tissue (VAT) (r = −0.43, p = 0.004) but not with the amount of liver fat (r = 0.22, p = 0.14).

**Fig. 2:**
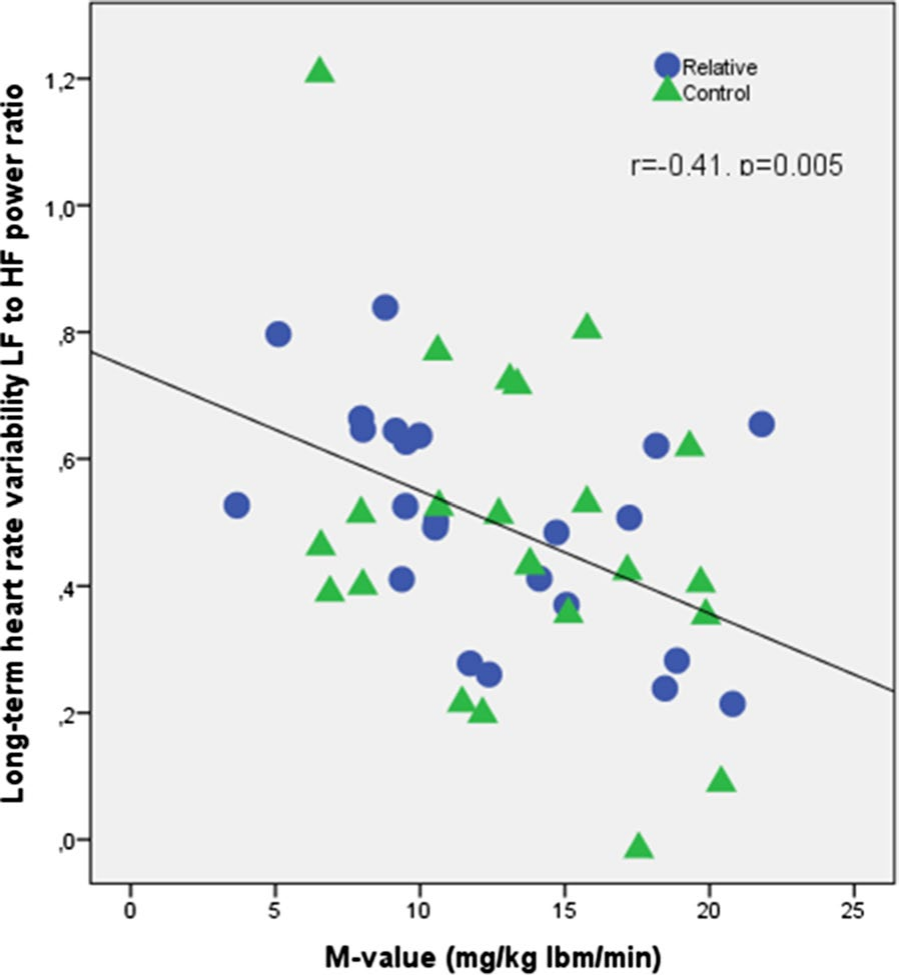
Long-term heart rate variability (HRV) assessed by 24-h ECG recordings. LF to HF power ratio was negatively and significantly associated with M value (mg/kg lbm/min) inrelatives (R = *blue circles*) and controls (C = *green triangles*) taken together

HF power and LF to HF power ratio in long-term HRV were significantly associated with M value (r = 0.30, p = 0.047 and r = −0.41, p = 0.005, respectively) and the inverse and significant association between LF to HF power ratio and M value is also displayed in Fig. 2. In addition, LF to HF power ratio was inversely associated with age (r = −0.40, p = 0.006) (not shown). HF power and SD1 were also significantly or near-significantly associated with M value in univariate analyses (r = 0.30, p = 0.047 and r = 0.29, p = 0.052, respectively) (Additional file 1: Table S2). We also evaluated the correlation between time- and frequency-domain variables in long-term HRV and a strong correlation was found between the power of the HF component and RMSSD (r^2^ = 0.88) and Ptot and SDNNindex (r^2^ = 0.94), respectively (not shown).

In a multivariate model BMI and LF to HF power ratio in long-term HRV but not age were significantly and independently associated with M value (r^2^ = 0.33, p = 0.001 for model, std β = −0.46, p < 0.001 and std β = −0.29, p = 0.04 and std β = 0.03, p = 0.81, respectively) (Table 3). When VAT was entered instead of BMI the model displayed a lower r^2^ (r^2^ = 0.26, p = 0.007 for model) but VAT and long-term LF to HF power ratio still remained significantly or near-significantly and independently associated with M value (std β = −0.35, p = 0.029 and std β = −0.31, of p = 0.054, respectively) (Table 3). Similar multivariate models using SD1 and HF power from long-term HRV were calculated but together they displayed a lower r^2^, and thus HF power and SD1 did not contribute significantly in the models (Additional file 1: Table S4). In summary, the best model to explain M value included BMI and LF to HF power ratio (std β = −0.46, p = 0.001 and std β = −0.28, p = 0.003; r^2^ = 0.37, p < 0.001 for model).

**Table 3:** Results from multiple linear regression analyses with insulin resistance (M value) as dependent variable and body composition (BMI or VAT), age, and LF-to-HF power ratio during long-term HRV entered as independent variables

Dependent variable	Independent variables	Std β	p value
M value	BMI	−0.46	0.001
	Age	0.03	0.81
	Long-term LF to HF power ratio	−0.29	0.040
M value	VAT	−0.35	0.029
	Age	0.06	0.04
	Long-term LF to HF power ratio	−0.31	0.054

Spectral indices are log-transformed. Age and body composition (BMI or VAT) were entered as independent variables

*HRV* heart rate variability, *LF to HF power ratio* ration between power of the low frequency and high frequency component, *BMI* body mass index, *VAT* visceral adipose tissue

### Short-term heart rate variability (HRV) and change in insulin sensitivity over time

Seven subjects had longitudinal data on short-term HRV and M value available at two time points mean 6 years apart. In these subjects, a high LF to HF power ratio at baseline was significantly associated with reduction in M value over time (r = −0.82, p = 0.024).

## Discussion

In the present study we show an association between insulin resistance and altered balance between sympathetic and parasympathetic nervous activity using long-term ECG registrations. In addition, we show differences in ANS balance between healthy subjects with either two first-degree or one first-degree plus at least two second-degree relatives with T2D (T2D relatives) and subjects without a history of T2D.

IR occurs early in the development of T2D and has been demonstrated already in healthy T2D relatives [1]. Previous studies have suggested that alteration of ANS activity is associated with and important for IR in vivo [6]. The findings in this study are in line with similar results obtained using short-term HRV assessments [9, 24] but this time was shown using long-term HRV assessments analysing indices from 24-h ECG-recordings.

Significant associations between IR (M value) and LF to HF power ratio in both short-term and long-term HRV imply and support that an imbalance in ANS regulation may play a role in IR. These associations remained significant or near-significant also after adjusting for both age and different aspects of body composition. The role of ANS dysregulation in the development of IR was further supported by a small subgroup analysis of seven subjects with available longitudinal data on HRV in whom LF to HF power ratio at baseline was significantly associated with change in insulin sensitivity (M value) during the following 6 years. An imbalance in the ANS could be explained by a combination of a relative increase in sympathetic activation as well as an attenuation of parasympathetic activity and could therefore be in line with previous findings that IR is associated with both a parasympathetic dysfunction as well as an enhanced sympathetic reactivity [25].

In the present study differences in HRV between T2D relatives and controls were more pronounced in long-term than the short-term assessments suggesting that other mechanisms, such as physical activity and other life-style factors also may be of importance [25]. In addition, age-adjusted total and very low frequency (VLF) power was significantly lower in T2D relatives indicating that reduced vagal activation potentially could be an early component in the development of T2D [26].

Obesity, perhaps via relative hyperinsulinemia, could be an additional link between IR and ANS activity since sympathetic activity is related to the degree of adiposity [8]. Our present study, in combination with previous work [8], support that obesity, and in particular visceral obesity, leads to dysregulated ANS activity, which in turn contributes to IR. However, somewhat surprisingly, acute pharmacological inhibition of vagal signals enhance insulin sensitivity in a previous study from our group [27]. Therefore it could be hypothesized that acute and chronic effects of the ANS could have differential impact on glucose metabolism. The finding that dietary intervention in obese subjects was followed by an increase in both vagal function and insulin sensitivity [28] also supports that IR may be modifiable by changes in the ANS.

The association between IR and visceral adiposity is well established [29, 30]. In this study VAT was associated with an increase in LF to HF power ratio and decreased HF power. When adjusting for VAT in analysis of short-term HRV parameters, only LF power and LF to HF power ratio during controlled breathing remained significantly associated with insulin sensitivity potentially indicating that IR is potentially more associated with parasympathetic dysregulation and visceral adiposity than sympathetic regulation. However, this study showed that models including BMI together with long-term HRV indices had the strongest association with IR indicating that total adiposity potentially may promote IR via an indirect increase in sympathetic/parasympathetic balance, for example via high FFA levels reaching the hypothalamus [31]. A recent report also showed that direct SNS innervation of adipocytes that can stimulate lipolysis [32], and thus possibly contributing to IR via elevated FFA levels [33]. Other factors like inflammation and leptin could be additional links between IR and ANS activity as indicated form other larger cohort studies [34, 35]. Another suggested link between IR and alterations in ANS activity, namely hyperinsulinemia, could potentially also be a link to hypertension, dyslipidemia and development of cardiovascular disease [36].

Life style factors, including stress, may also be of importance and it is therefore interesting that pronounced differences in HRV were found between the T2D relatives and controls analysing the 24-h ECG-recordings during normal activities supporting an attenuation of the parasympathetic activity in T2D relatives when compared to control subjects. Since no significant differences in metabolic parameters, body composition or the Everyday Life Stress Scale instrument were found between the two groups this may suggest an underlying (epi) genetic difference in autonomic nerve activity that precedes development of IR. This is in agreement with clinical longitudinal studies [37, 38].

Many of the time- and frequency-domain variables measured over the entire 24 h period are strongly correlated with each other [39]. The advantage of frequency-domain analysis, as used in this study, is the association between spectral components and the activity in the specific parts of the autonomic system. LF to HF power ratio, considered to reflect sympathetic nerve activity, cannot be assessed using time-domain analyses.

A weakness of our study is that no formal power calculation was performed and that the study has limited power in analysing the relationship between IR and activity of the ANS. It must also be appreciated that the activity of the ANS was only examined at the level of the heart, which might differ from the ANS activity in other organs crucial for insulin action such as liver, muscle and adipose tissue.

In conclusion, the present study shows that indices of heart rate variability during everyday life are associated with insulin sensitivity, and it suggests that a higher ratio of sympathetic to parasympathetic autonomic nerve activity promotes insulin resistance. Longitudinal data from a subgroup analysis support that such an imbalance in the ANS may precede development of insulin resistance. Moreover, first-degree relatives of type 2 diabetes patients had reduced total heart rate variability and an elevated heart rate suggesting that certain genetic or epigenetic factors may contribute both to dysregulation of ANS and potentially to the development of type 2 diabetes.

## Additional file

10.1186/s12933-016-0411-8 HRV parameters from the short-term recordings. **Table S2.** Univariate associations between IR and long-term HRV frequency domain variables. **Table S3.** Results from multiple linear analyses with IR and age, body composition and other long-term HRV variables. **Table S4.** Multivariate associations between IR and long-term HRV adjusting for age and body composition.

## Abbreviations

HR: heart rate
HRV: heart rate variability
*P*_*tot*_: total spectral power
*P*_*LF*_: spectral power of the low-frequency component
*P*_*HF*_: spectral power of the high-frequency component
OGTT: oral glucose tolerance test
IGT: impaired glucose tolerance
CPT: cold pressor test
NEFA: non-esterified fatty acids
IR: insulin resistance
ISI: insulin sensitivity index
T2D: type 2 diabetes

## References

[1] Eriksson J, Franssila-Kallunki A, Ekstrand A (1989). Early metabolic defects in persons at increased risk for non-insulin-dependent diabetes mellitus. N Engl J Med.

[2] Vestergaard H, Bjorbaek C, Andersen PH, Bak JF, Pedersen O (1991). Impaired expression of glycogen synthase mRNA in skeletal muscle of NIDDM patients. Diabetes.

[3] Beck-Nielsen H, Groop LC (1994). Metabolic and genetic characterization of prediabetic states. Sequence of events leading to non-insulin-dependent diabetes mellitus. J Clin Invest.

[4] Zierath JR, Galuska D, Nolte LA, Thorne A, Kristensen JS, Wallberg-Henriksson H (1994). Effects of glycaemia on glucose transport in isolated skeletal muscle from patients with NIDDM: in vitro reversal of muscular insulin resistance. Diabetologia.

[5] Buren J, Lindmark S, Renstrom F, Eriksson JW (2003). In vitro reversal of hyperglycemia normalizes insulin action in fat cells from type 2 diabetes patients: is cellular insulin resistance caused by glucotoxicity in vivo?. Metabolism.

[6] Huggett RJ, Hogarth AJ, Mackintosh AF, Mary DA (2006). Sympathetic nerve hyperactivity in non-diabetic offspring of patients with type 2 diabetes mellitus. Diabetologia.

[7] Thorp AA, Schlaich MP (2015). Relevance of sympathetic nervous system activation in obesity and metabolic syndrome. J Diabetes Res.

[8] Lindmark S, Lonn L, Wiklund U, Tufvesson M, Olsson T, Eriksson JW (2005). Dysregulation of the autonomic nervous system can be a link between visceral adiposity and insulin resistance. Obes Res.

[9] Laitinen T, Vauhkonen IK, Niskanen LK (1999). Power spectral analysis of heart rate variability during hyperinsulinemia in nondiabetic offspring of type 2 diabetic patients: evidence for possible early autonomic dysfunction in insulin-resistant subjects. Diabetes.

[10] Saito I, Hitsumoto S, Maruyama K (2015). Heart rate variability, insulin resistance, and insulin sensitivity in Japanese adults: The Toon Health Study. J Epidemiol.

[11] Stuckey MI, Kiviniemi A, Gill DP, Shoemaker JK, Petrella RJ (2015). Associations between heart rate variability, metabolic syndrome risk factors, and insulin resistance. Appl Physiol Nutr Metab.

[12] Wulsin LR, Horn PS, Perry JL, Massaro J, D’Agostino R. Autonomic imbalance as a predictor of metabolic risks, cardiovascular disease, diabetes, and mortality autonomic imbalance predicts CVD, DM, Mortality. J Clin Endocrinol Metab. jc20144123. 2015. 10.1210/jc.2015-1748 26047073

[13] Malliani A, Lombardi F, Pagani M (1994). Power spectrum analysis of heart rate variability: a tool to explore neural regulatory mechanisms. Br Heart J.

[14] Lukaski HC (1997). Validation of body composition assessment techniques in the dialysis population. ASAIO J.

[15] Claesson M, Burell G, Birgander LS, Lindahl B, Asplund K (2003). Psychosocial distress and impaired quality of life–targets neglected in the secondary prevention in women with ischaemic heart disease. Eur J Cardiovasc Prev Rehabil.

[16] Eriksson JW, Jansson PA, Foley K, Lithell H (1996). Insulin sensitivity following treatment with the alpha 1-blocker bunazosin retard and the beta 1-blocker atenolol in hypertensive non-insulin-dependent diabetes mellitus patients. J Hypertens.

[17] Chowdhury B, Sjostrom L, Alpsten M, Kostanty J, Kvist H, Lofgren R (1994). A multicompartment body composition technique based on computerized tomography. Int J Obes Relat Metab Disord.

[18] Rasband WS. ImageJ (version v1.37s). US National Institutes of Health, Bethesda. 2006. http://rsb.info.nih.gov/ij/. Assessed 17 June 2016.

[19] DeFronzo R, Deibert D, Hendler R, Felig P, Soman V (1979). Insulin sensitivity and insulin binding to monocytes in maturity-onset diabetes. J Clin Invest.

[20] Blom H, Andersson C, Olofsson BO, Bjerle P, Wiklund U, Lithner F (1996). Assessment of autonomic nerve function in acute intermittent porphyria; a study based on spectral analysis of heart rate variability. J Intern Med.

[21] Wiklund U, Hornsten R, Karlsson M, Suhr OB, Jensen SM (2008). Abnormal heart rate variability and subtle atrial arrhythmia in patients with familial amyloidotic polyneuropathy. Ann Noninvasive Electrocardiol.

[22] Karlsson M, Hornsten R, Rydberg A, Wiklund U (2012). Automatic filtering of outliers in RR intervals before analysis of heart rate variability in Holter recordings: a comparison with carefully edited data. Biomed Eng Online.

[23] Luutonen S, Antila K, Neuvonen P, Raiha I, Rajala T, Sourander L (1994). Spectral analysis of heart rate variability in evaluation of sympathetic function in elderly subjects. Age Ageing.

[24] Lindmark S, Wiklund U, Bjerle P, Eriksson JW (2003). Does the autonomic nervous system play a role in the development of insulin resistance? A study on heart rate variability in first-degree relatives of type 2 diabetes patients and control subjects. Diabet Med.

[25] Foss CH, Vestbo E, Froland A, Gjessing HJ, Mogensen CE, Damsgaard EM (2001). Autonomic neuropathy in nondiabetic offspring of type 2 diabetic subjects is associated with urinary albumin excretion rate and 24-h ambulatory blood pressure: the Fredericia study. Diabetes.

[26] Tripathi KK (2011). Very low frequency oscillations in the power spectra of heart rate variability during dry supine immersion and exposure to non-hypoxic hypobaria. Physiol Meas.

[27] Svensson MK, Jansson PA, Persson AL, Sjostrand M, Eriksson JW (2011). Atropine improves insulin sensitivity in both lean and abdominally obese subjects. J Clin Endocrinol Metab.

[28] Ziegler D, Strom A, Nowotny B (2015). Effect of low-energy diets differing in fiber, red meat, and coffee intake on cardiac autonomic function in obese individuals with type 2 diabetes. Diabetes Care.

[29] Hansen T, Ahlstrom H, Soderberg S (2009). Visceral adipose tissue, adiponectin levels and insulin resistance are related to atherosclerosis as assessed by whole-body magnetic resonance angiography in an elderly population. Atherosclerosis.

[30] Preis SR, Massaro JM, Robins SJ (2010). Abdominal subcutaneous and visceral adipose tissue and insulin resistance in the Framingham heart study. Obesity (Silver Spring).

[31] Benthem L, Keizer K, Wiegman CH (2000). Excess portal venous long-chain fatty acids induce syndrome X via HPA axis and sympathetic activation. Am J Physiol Endocrinol Metab.

[32] Zeng W, Pirzgalska RM, Pereira MM (2015). Sympathetic neuro-adipose connections mediate leptin-driven lipolysis. Cell.

[33] Boden G (1999). Free fatty acids, insulin resistance, and type 2 diabetes mellitus. Proc Assoc Am Physicians.

[34] Saito I, Hitsumoto S, Maruyama K (2016). Impact of heart rate variability on C-reactive protein concentrations in Japanese adult nonsmokers: The Toon Health Study. Atherosclerosis.

[35] Kurajoh M, Koyama H, Kadoya M (2015). Plasma leptin level is associated with cardiac autonomic dysfunction in patients with type 2 diabetes: HSCAA study. Cardiovasc Diabetol.

[36] Ginsberg HN (2000). Insulin resistance and cardiovascular disease. J Clin Invest.

[37] Licht CM, de Geus EJ, Penninx BW (2013). Dysregulation of the autonomic nervous system predicts the development of the metabolic syndrome. J Clin Endocrinol Metab.

[38] Jiang X, Liu X, Wu S (2015). Metabolic syndrome is associated with and predicted by resting heart rate: a cross-sectional and longitudinal study. Heart.

[39] Heart rate variability. Standards of measurement, physiological interpretation, and clinical use. Task Force of the European Society of Cardiology and the North American Society of Pacing and Electrophysiology. Eur Heart J. 1996;17:354–81. 8737210

