# Supplementary material for: Alterations in heart rate variability during everyday life are linked to insulin resistance. A role of dominating sympathetic over parasympathetic nerve activity?
Source: Cardiovasc Diabetol. 2016 Jun 28;15:91. doi: 10.1186/s12933-016-0411-8 (PMC4924321; doi:10.1186/s12933-016-0411-8)
Supplement: Supplementary file 1 — 10.1186/s12933-016-0411-8 HRV parameters from the short-term recordings. Table S2. Univariate associations between IR and long-term HRV frequency domain variables. Table S3. Results from multiple linear analyses with IR and age, body composition and other long-term HRV variables. Table S4. Multivariate associations between IR and long-term HRV adjusting for age and body composition. [file 12933_2016_411_MOESM1_ESM.docx]

**Supplementary tables**

**Table S1**. Heart rate variability (HRV) parameters from the short-term recordings during manoeuvres that activate parasympathetic (controlled breathing) or the sympathetic (tilt and cold pressor test) automatic nervous system in T2D relatives (n=23) and control subjects (n=24).

|  |  | **Supine** | **Controlled**  **breathing** | **Upright**  **tilt** | **Cold**  **provocation** | **p-value** |
| --- | --- | --- | --- | --- | --- | --- |
| *P_HF_ (ms^2^, log)* | Relatives | 2.65 (0.48) | 2.91 (0.56) | 2.34 (0.57) | 2.75 (0.40) |  |
|  | Controls | 2.61 (0.61) | 2.95 (0.64) | 2.53 (0.66) | 2.91 (0.55) | 0.19 |
|  |  |  |  |  |  |  |
| *P_LF_/P_HF_ (log)* | Relatives | 0.16 (0.43) | -0.25 (0.39) | 0.83 (0.39) | 0.34 (0.28) |  |
|  | Controls | 0.13 (0.48) | -0.44 (0.50) | 0.69 (0.59) | 0.17 (0.41) | 0.11 |

Values are mean and (sd). Spectral indices are log-transformed. P-values are derived from a comparison of relatives and controls analysis of variance of repeated measurements (ANCOVA), adjusting for age.

HRV=heart rate variability; P_HF_=power of high frequency component; P_LF_/P_HF_ = ratio of power of low frequency to power of high frequency.

**Table S2**. Univariate associations between insulin resistance (M-value) and long-term HRV frequency domain variables and Poincaré indices.

| **Long-term HRV variables** | **r** | **p-value** |
| --- | --- | --- |
| SD1 | 0.29 | **0.052** |
| SD2 | 0.24 | 0.11 |
| SD1 to SD 2 ratio | 0.24 | 0.10 |
| RR interval | 0.08 | 0.60 |
| HRV total power | 0.13 | 0.39 |
| VLF power | 0.12 | 0.44 |
| LF power | 0.06 | 0.69 |
| HF power | 0.30 | 0.047 |
| LF to HF power ratio | -0.41 | 0.005 |

Long-term HRV parameters with p-value ≤0.1 are shown (see methods section). Spectral indices are log-transformed. HRV=heart rate variability; SD1=Magnitude of the beat-to-beat variability over the 24-hour period; SD2=Fluctuations in mean RR interval over the 24-hour period; RR interval=mean RR interval (s) (average heart rate); HRV total power=power of total HRV; VLF power=power of very low frequency component; LF power=power of low frequency component; HF power=power of high frequency component; LF to HF power ratio=ratio between power of low frequency and power of high frequency component.

**Table S3.** Results from multiple linear analyses with insulin resistance (M-value) as dependent variable and age, body composition (BMI or VAT) and other long-term HRV variables than long-term LF-to-HF power ratio (HF power and SD1) entered as independent variables.

| **Dependent variable** | **Independent variables** | **Std β** | **p-value** |
| --- | --- | --- | --- |
| **M-value**  (r^2^ =0.33, p=0.001 for model) | BMI | -0.50 | <0.001 |
|  | Age | 0.06 | 0.73 |
|  | Long-term HF power | 0.22 | 0.19 |
| **M-value**  (r^2^ =0.24, p=0.013 for model) | VAT | -0.41 | 0.012 |
|  | Age | 0.13 | 0.50 |
|  | Long-term HF power | 0.29 | 0.12 |
| **M-value**  (r^2^ =0.32, p=0.001 for model) | BMI | -0.50 | <0.001 |
|  | Age | 0.02 | 0.90 |
|  | SD1 | 0.18 | 0.26 |
| **M-value**  (r^2^ =0.22, p=0.021 for model) | VAT | -0.41 | 0.014 |
|  | Age | 0.07 | 0.72 |
|  | SD1 | 0.21 | 0.23 |
| Spectral indices are log-transformed. Age and body composition (BMI or VAT) are entered as independent variables. HRV=heart rate variability; HF power=power of high frequency component, SD1= Magnitude of the beat-to-beat variability over the 24-hour period. | | | |

**Table S4.** Multivariate associations between insulin resistance (M-value) and long-term heart rate variability and adjusting for age and body composition (BMI or VAT).

| **Dependent variable** | **Independent variables adjusting for age and *BMI*** | **Std β** | **p-value** |
| --- | --- | --- | --- |
| *M-value* | Long-term LF to HF power ratio | -0.29 | 0.04 |
|  |  | | |
|  | **Independent variables adjusting for age and *VAT*** | | |
|  | Long-term LF to HF power ratio | -0.31 | 0.054 |
|  | | | |
| *Long-term LF to HF power ratio* | **Independent adjusting for age and BMI** |  |  |
|  | M-value | -0.33 | 0.04 |
|  |  | | |
|  | **Independent adjusting for age and VAT** | | |
|  | M-value | -0.30 | 0.054 |
| Spectral indices are log-transformed. HRV=heart rate variability; LF to HF power ratio=ratio between power of low frequency and power of high frequency component. BMI=body mass index; VAT=visceral adipose tissue. Adjustments for age and body composition (BMI or VAT) as indicated | | | |
